# Polydatin: A Critical Promising Natural Agent for Liver Protection via Antioxidative Stress

**DOI:** 10.1155/2022/9218738

**Published:** 2022-02-10

**Authors:** Dandan Tang, Qing Zhang, Huxinyue Duan, Xun Ye, Jia Liu, Wei Peng, Chunjie Wu

**Affiliations:** School of Pharmacy, Chengdu University of Traditional Chinese Medicine, No. 1166, Liutai Avenue, Chengdu 611137, China

## Abstract

Polydatin, one of the natural active small molecules, was commonly applied in protecting and treating liver disorders in preclinical studies. Oxidative stress plays vital roles in liver injury caused by various factors, such as alcohol, viral infections, dietary components, drugs, and other chemical reagents. It is reported that oxidative stress might be one of the main reasons in the progressive development of alcohol liver diseases (ALDs), nonalcoholic liver diseases (NAFLDs), liver injury, fibrosis, hepatic failure (HF), and hepatocellular carcinoma (HCC). In this paper, we comprehensively summarized the pharmacological effects and potential molecular mechanisms of polydatin for protecting and treating liver disorders via regulation of oxidative stress. According to the previous studies, polydatin is a versatile natural compound and exerts significantly protective and curative effects on oxidative stress-associated liver diseases via various molecular mechanisms, including amelioration of liver function and insulin resistance, inhibition of proinflammatory cytokines, lipid accumulation, endoplasmic reticulum stress and autophagy, regulation of PI3K/Akt/mTOR, and activation of hepatic stellate cells (HSCs), as well as increase of antioxidant enzymes (such as catalase (CAT), glutathione peroxidase (GPx), glutathione (GSH), superoxide dismutase (SOD), glutathione reductase (GR), and heme oxygenase-1 (HO-1)). In addition, polydatin acts as a free radical scavenger against reactive oxygen species (ROS) by its phenolic and ethylenic bond structure. However, further clinical investigations are still needed to explore the comprehensive molecular mechanisms and confirm the clinical treatment effect of polydatin in liver diseases related to regulation of oxidative stress.

## 1. Introduction

Increasing epidemic investigations have suggested that liver diseases remain one of the leading causes of deaths globally, and millions of people are suffering from acute or chronic liver disorders nowadays [1]. Currently, the morbidity of metabolic liver diseases including nonalcoholic fatty liver disease (NAFLD) and alcohol liver disease (ALD) are rising rapidly due to the continuous improving living standards, and it is reported that more than 10% of the world population were affected by liver diseases. Furthermore, the NAFLD and ALD are the very serious factors for ultimately leading to more cases of end-stage liver diseases, including hepatic failure (HF), cirrhosis, and hepatocellular carcinoma (HCC) [2].

Oxidative stress is a state due to the imbalance of free radicals and antioxidative enzymes. The imbalance tends to be oxidized, which leads to inflammatory infiltration of neutrophils, increasing the secretion of proteases and large amounts of oxidative intermediate products. Oxidative stress is produced by the excessive free radicals in the body which is considered as one of the most important factors to aging and various diseases. Redox state constitutes a necessary background of multiple liver diseases [3]. Oxidative stress is an important factor for development of liver diseases, especially in chronic liver diseases [4], and oxidative stress-associated liver diseases could also result in kidney injury and brain impairment [5, 6]. Reactive oxygen species (ROS), a highly reactive species of free radical, plays dual roles in living systems [7]. At physiological concentration, ROS plays essential roles in physiological process such as gene expression, signal transduction, and redox regulation. However, during some pathological conditions, the excessive ROS production has harmful effects for human body, such as damages of proteins, DNA, and lipids [8]. In addition, many etiological factors associated with liver disease are commonly highly productive under excessive ROS. It is reported that mitochondrial ROS levels could be highly increased by ROS, reactive nitrogen species (RNS), and excessive alcohol consumption in hepatocytes [9, 10]. Oxidative stress might be one of the main reasons in the progressive development of alcohol liver diseases (ALDs), nonalcoholic liver diseases (NAFLDs), liver injury, fibrosis, hepatic failure (HF), and hepatocellular carcinoma (HCC). Detection redox biomarkers may help diagnose liver diseases. For example, Świderska et al. found that advanced glycation end products (AGEs), an oxidative damage product, maybe a potential biomarker in NAFLD diagnostics [11].

**Figure 1:**
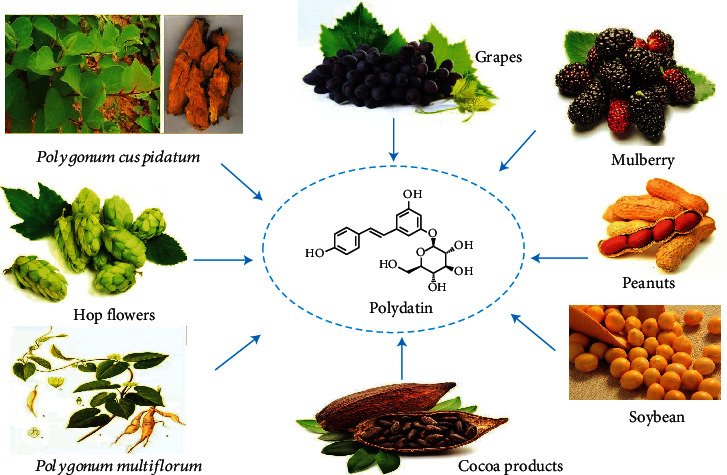
The plant sources of PD.

Accumulating researches have shown that natural activity compounds such as paeoniflorin, taraxasterol, and oxymatrine possess versatile advantages for treating liver diseases with low toxicity and reliable pharmacological activities. Therefore, in the Europe and United States, approximately 65% of patients would like to use herbal medicines to treat liver diseases [12–15]. Polydatin (3,5,4-trihydroxystilbene-3-*O*-*β*-*D*-glucopyranoside, PD), commonly isolated from the roots of *Polygonum cuspidatum*, can be also obtained from many dietary supplements like grapes, peanuts, cocoa products, hop flowers (*Humulus lupulus*), and other plants (Figure 1 and Table 1) [16–21]. Preclinical trials revealed that PD has various pharmacological activities, such as anti-inflammatory [22–24], antiapoptotic [25], antitumor [26], lipid-lowering [27], and cardiovascular protection effects [28–30], especially exhibited strengthened pharmacological activities in antioxidant. The antioxidant activities were involved in immune system, osteoarthritis, endometriosis, pain, and intestinal inflammation and reported to be effective in treating liver disorders [31–35]. In the present review, we summarized and discussed the versatile effects of PD against liver diseases via regulation of oxidative stress.

## 2. Effects of Polydatin on Liver Diseases via Regulating Oxidative Stress

**Figure 2:**
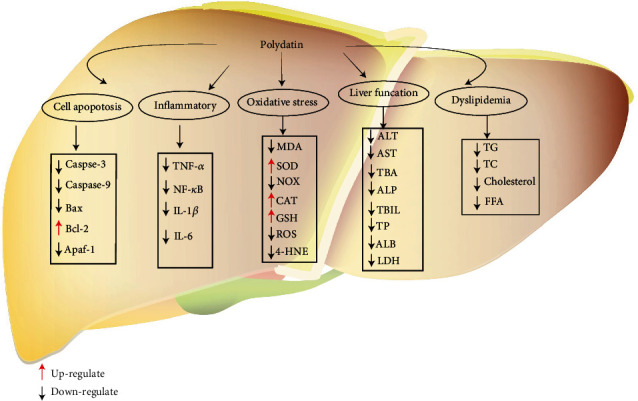
Cellular and molecular mechanisms of PD in the prevention of oxidative stress induced liver diseases. Bax: BCL-2-associated; Bcl-2: B-cell lymphoma-2; MDA: malondialdehyde; SOD: superoxide dismutase; NOX: nicotinamide adenine dinucleotide phosphate oxidative; CAT: catalase; GSH: glutathione; ROS: reactive oxygen species; 4-HNE: 4-hydroxynonenal; TNF-*α*: tumor necrosis factor-*α*; NF-*κ*B: nuclear factor kappa B; IL-1*β*: interleukin-1*β*; IL-6: interleukin-6; ALT: alanine aminotransferase; AST: aspartate aminotransferase; TBA: total bile acid; TBIL: total bilirubin; ALP: alkaline phosphatase; ALB: albumin; LDH: lactate dehydrogenase; TG: triglyceride; TC: total cholesterol; FFA: free fatty acid.

By collecting relevant literatures in the past decades regarding the protection and treatment of PD against liver diseases, it has been found that PD has marked effects on liver-related conditions, including nonalcoholic fatty liver disease (NAFLD), alcohol liver disease (ALD), liver fibrosis, and HCC. The specific molecular mechanisms of protective and therapeutic effects of PD are concluded in Figure 2 and Table 2.

### 2.1. Polydatin and Alcoholic Liver Diseases

**Figure 3:**
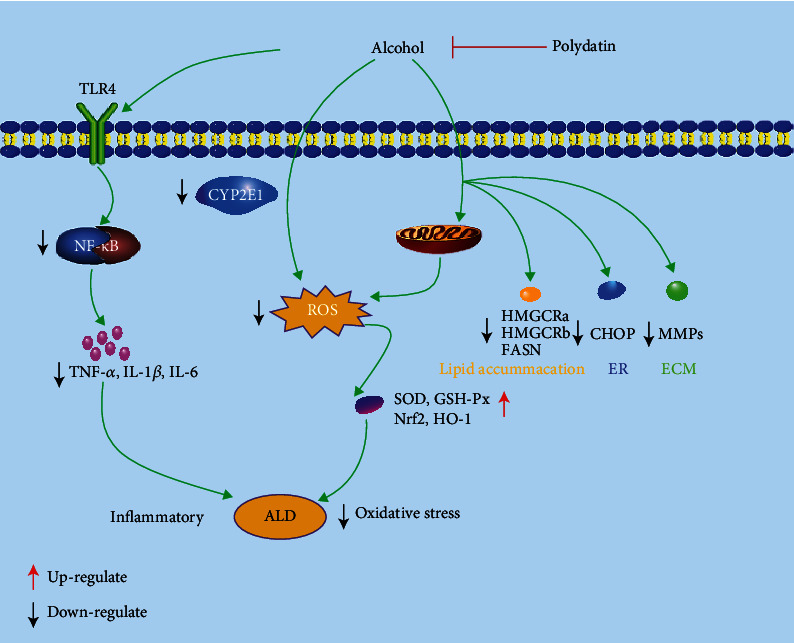
Cellular and molecular mechanisms of PD in the prevention of oxidative-associated alcoholic liver disease.

In the past 30 years, alcohol consumption has a dramatically increased tendency due to the booming economy in China, leading to the highly incidence of alcohol liver disease (ALD) at 4.5% [36]. Nowadays, ALD has become one of the leading causes of chronic diseases and death in the world. Therefore, previous researchers paid much attention to ALD due to the molecular mechanism of ALD not completely clear [37]. ALD covers a wide spectrum of histological features, ranging from lipid accumulation in liver cells (fatty liver or steatosis) with minimal parenchymal damage to more severe liver injury, including steatohepatitis fibrosis/cirrhosis [38]. It was widely accepted that ALD pathogens are related to lipid accumulation, oxidative stress, inflammation, and mitochondrial dysfunction (Figure 3). Among the pathogenesis of ALD, oxidative stress and inflammation were considered as the fundamental mechanisms [39]. Alcohol-induced liver damage is related to excessive production of ROS and the presence of oxidative stress in liver cells. About 90% of alcohol was metabolized in the liver. Some metabolic enzymes in the liver, including alcohol dehydrogenase (ADH) and cytochrome P4502E1 (CYP2E1), converted alcohol to acetaldehyde. Then, acetaldehyde was oxidized to acetate by aldehyde dehydrogenase (ALDH) and converted to carbon dioxide through the citric acid cycle [40]. The main resource of ROS in the liver is related to the cytochrome P450 enzymes. Using the liver-injured male rats induced by ethanol, pretreatment with PD could improve the liver injury via suppressing oxidative stress by upregulation of ADH and ALDH and downregulating CYP2E1 [41]. Hepatic steatosis model was established in zebrafish induced by ethanol larvae, and it is found that PD treatment could improve ethanol metabolism by decreasing the gene expressions of CYP2Y3 and CYP3A [42]. Furthermore, the capacity of alcohol could stimulate the production of free radicals, which impaired liver antioxidant defense capability and greatly promoted the oxidative stress damage of ALD [38]. PD could improve the activities of glutathione peroxidase (GSH-Px), superoxide dismutase (SOD), and upregulated nuclear factor erythroid 2-related factor 2 (Nrf2) and its target gene heme oxygenase-1 (HO-1) [41]. Besides, oxidative stress might contribute to the pathogenesis of alcoholic steatosis through actions on transcription factors regulating mitochondrial injury, lipid metabolism, DNA damage, and endoplasmic reticulum (ER) stress. PD treatment could decrease the mRNA levels of fatty acid synthase (FASN), HMGCRa, and HMGCRb to attenuate hepatic fat accumulation [42]. Moreover, DNA damage and ER stress play a key role in the disruption of lipid homeostasis, metabolism, and liver function [43]. PD could improve ethanol-induced DNA damage and ER stress by decreasing the mRNA levels of C/EBP homologous protein (CHOP) and growth arrest and DNA damage-inducible gene, 45*α*a (GADD45*α*a) in zebrafish larvae [42]. Acetaldehyde is a vital metabolite of alcohol and ROS, which can stimulate the secretion of matrix metalloproteinase (MMP). MMP promotes the degradation of extracellular matrix (ECM) components while distorting liver tissue structure [45]. Pretreatment with PD at the doses 50 and 100 mg/kg, respectively, can significantly prevent the rise in MMP activities in the liver tissue [46]. Furthermore, mitochondria are highly sensitive to oxidative stress. ROS accumulation in the mitochondrial membrane will deplete the mitochondrial complexes and cause mitochondrial dysfunctions including deterioration of respiratory enzymes, enhanced mitochondrial stress, and loss of functioning in mitochondria. Mitochondrial dysfunctions may eventually lead to apoptosis or necrotic cell death in liver tissue [44, 47]. Pretreatment with PD could ameliorate the activities of redox and mitochondrial respiratory enzyme, such as succinate dehydrogenase, NADH dehydrogenase, and cytochrome c (Cyt-C) oxidase. It is reported that PD could restore the mitochondrial respiratory complexes and ameliorate their functioning, providing evidence for its hepatoprotective potential through the mitochondrial oxidative stress inhibitory activity [46].

There is increasing evidence that long-term excessive alcohol intaking can increase the release of inflammatory cytokines [48]. PD treatment decreased the levels of proinflammatory cytokines including interleukin-6 (IL-6), interleukin-1*β* (IL-1*β*), and tumor necrosis factor-*α* (TNF-*α*) through downregulating toll-like receptor 4 (TLR4) and nuclear factor kappa B (NF-*κ*B) p65 [41]. Besides, PD could improve the liver function by decreasing the levels of lactate dehydrogenase (LDH), aspartate aminotransferase (AST), alanine aminotransferase (ALT), and alkaline phosphatase (ALP) in the serum [41, 46].

To conclude, previous studies indicated that pretreatment of PD could alleviate liver diseases induced by alcohol. PD exerts its protective activity by resisting the oxidative stress induced by alcohol and restoring the antioxidant balance and the MMP/TIMP ratio of hepatic tissue.

### 2.2. Polydatin and Nonalcoholic Fatty Liver Diseases

**Figure 4:**
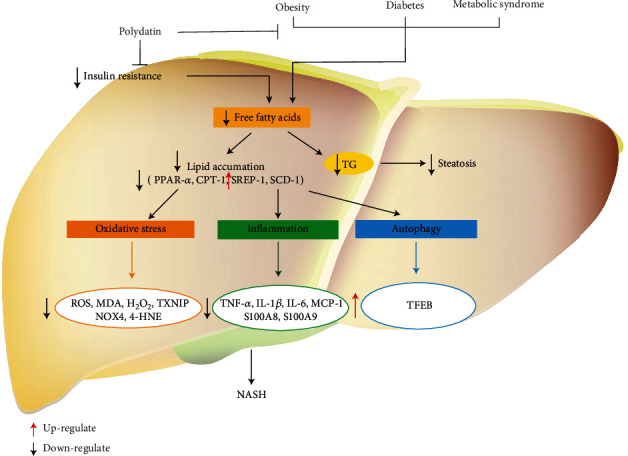
Cellular and molecular mechanisms of PD in the prevention of oxidative-associated nonalcoholic liver diseases.

Nowadays, nonalcoholic fatty liver disease (NAFLD) is emerging as one of the most common causes of chronic liver disease due to the increasing incidence of obesity, diabetes, and metabolic syndrome in the general population [49, 50]. NAFLD had affected about 173 million to 338 million people in China, and its prevalence was estimated by 25.2% in the world [36]. NAFLD encompasses a broad spectrum of liver injury ranging from simple triglyceride (TG) accumulation in the liver (steatosis) to nonalcoholic steatohepatitis (NASH), which may lead to fibrosis and cirrhosis [51]. The pathogens of NAFLD involve in lipid accumulation, oxidative stress, and inflammation (Figure 4). It was reported that the first stage of NAFLD was lipid accumulation in the hepatocytes [52]. In normal circumstances, insulin inhibits adipose tissues releasing free fatty acid (FFA). However, with the development of insulin resistance, the increased plasma concentrations of glucose and fatty acids promote hepatic fatty acid synthesis and damage *β*-oxidation, leading to hepatic steatosis. Hepatic steatosis conversely exacerbates the degree of insulin resistance and accelerates the subsequent transition to steatohepatitis and fibrosis [53, 54]. Supplemented with PD for 12 weeks in methionine- and choline-deficient- (MCD-) induced model rats can alleviate the insulin resistance and improve basal insulin resistance values and glucose tolerance test in homeostasis model assessment. Besides, abnormal adiponectin and leptin levels were also corrected by PD supplementation. Additionally, PD could enhance insulin sensitivity via upregulating expression levels of insulin receptor substrate 2 and Akt phosphorylation in the rat liver induced by a high-fat diet (HFD) [55]. Besides, PD abrogated slight liver steatosis, increased carnitine palmitoyl transferase-1 (CPT-1) and peroxisome proliferator-activated receptor-*α* (PPAR-*α*) protein levels, decreased stearoyl-CoA desaturase-1 (SCD-1) and sterol regulatory element binding protein 1 (SREBP-1) protein levels, and reduced total cholesterol (TC) and TG levels in the fructose-fed liver of rats [56]. These results demonstrated that PD could inhibit hepatosteatosis via the reduction of the lipid accumulation.

In NAFLD pathogenesis, oxidative stress is considered as a vital factor [57]. It is reported that PD could alleviate liver oxidative stress *in vivo* and *in vitro*. *In vivo*, PD reduced the levels of malondialdehyde (MDA), ROS, and hydrogen peroxide (H_2_O_2_) and decreased thioredoxin-interacting protein (TXNIP) at concentrations of 7.5-30 mg/kg. *In vitro*, PD could reduce the levels of ROS and TXNIP and enhance miR-200a targeting Keap1/Nrf2 pathway in fructose-induced HepG2 and BRL-3A cells [56]. In pathological conditions, ROS overproduction was induced by nicotinamide adenine dinucleotide phosphate (NADPH) oxidative (NOX). In the NOX family, abnormal expression of NOX4 has been implicated in mice with diet-induced steatohepatitis and patients with NASH related to oxidative stress [58]. Intraperitoneally injected with 5 mg/kg PD reduced oxidative stress by decreasing the levels of NOX4, ROS, and 4-hydroxynonenal (4-HNE) in MCD-induced NASH C57BL/6 mice [59].

Apart from lipid accumulation and oxidative stress, inflammation also plays a crucial role in the development of NAFLD. Excessive fructose and HFD consumption cause NAFLD pathogenesis. PD could downregulate apoptosis-associated speck-like protein (ASC), and the NOD-like receptor family, pyrin domain containing 3 (NLRP3) protein levels and IL-1*β* were released in the liver of fructose-induced rats [56]. In another study, it was reported that PD treatment for 4 weeks can remarkably reduce Gr-1^+^ cells and alleviate hepatocyte steatosis and decrease expressions of proinflammatory factors including S100A8, S100A9, and monocyte chemoattractant protein-1 (MCP-1) in the liver tissues of HFD mice [60, 61]. Besides, treatment with PD reduced mRNA levels of proinflammatory cytokines such as IL-6, TNF-*α*, and CD68 macrophage activation related to the suppression of toll-like receptor (TLR) 4/NF-*κ*B p65 signaling pathway [58, 60].

Autophagy is a system which could regulate intracellular degradation. The development of NASH is considered to be related to impaired autophagic degradation of intracellular lipids. Autophagy regulates lipid metabolism and insulin resistance in the liver and protects hepatocytes from injury and cell death [62]. *In vivo*, oral administration of 100 mg/kg PD decreased hepatic lipid accumulation and alleviated inflammation and hepatocyte injury in MCD-induced db/db mice. *In vitro*, PD reduced palmitic acid-induced lipid accumulation in cultured hepatocytes. Both *in vivo* and *in vitro*, PD could restore lysosomal function and autophagic flux which was damaged by steatosis or NASH. In conclusion, PD inhibited PI3K/Akt/mTOR signaling pathway and increased the expression and activity of transcription factor EB (TFEB), a known master regulator of lysosomal function [63].

### 2.3. Polydatin and Liver Injury and Fulminant Hepatic Failure

Currently, lots of the commonly used drugs, including analgesic, anticancer drugs, agent antiphlogistic, and antidepressant, might be hepatotoxicity for human being [64]. Acetaminophen (APAP), a commonly used drug in clinical, was applied for ameliorating fever and pain. At standard doses, APAP exerts remarkable healing effects; however, when taken in overdose amounts, it could initiate acute hepatotoxicity and hepatic injury [65]. In addition, PD was found to show protective effect against APAP-induced hepatotoxicity via improving liver functions, alleviating oxidative stress, and suppressing apoptosis. Pretreatment of PD for 7 days at the doses of 25-100 mg/kg could effectively increase the survival rate of APAP-treated mice, significantly relieve histopathologic alterations in liver, and decrease the levels of AST and ALT in serum. Besides, PD treatment markedly and dose-dependently decreased oxidative stress by decreasing the levels of ROS, MDA, nitric oxide (NO), and GSSG and increasing the liver activities of GSH-Px, GSH, and the GSH/GSSG. Meanwhile, iNOS and NOX2 were also inhibited by PD treatment. Additionally, it is reported that PD significantly inhibited apoptosis of hepatocytes via increasing Bcl-2 and decreasing Bax, Apaf-1, Cyt-C, cleaved- (C-) caspase-9, and C-caspase-3 [66]. In another study, treatment with PD at the doses of 25-100 mg/kg/day has significant protective effect against cisplatin-induced oxidative stress and enhances antioxidant defense enzymes in mice [67].

Sulfur mustard (SM), a chemical warfare agent applied in a series of military conflicts, possesses serious threat to civilians and military soldiers [68]. Although the molecular mechanisms of SM induced hepatotoxicity were still unclear, it is recognized that oxidative stress plays predominant roles in the SM-induced liver damage [69]. PD treatment could dramatically increase the survival rate of mice with subcutaneously injection of SM. Additionally, PD treatment decreased the serum aminotransferase and alleviated SM-induced liver damage in mice. What is more, PD can also remarkably upregulate sirtuin-1 (Sirt1), NAD(P)H, quinone oxidoreductase-1 (NQO1), and Nrf2 and HO-1 in L02 cells and liver tissues of mice [70]. CCl_4_ can cause severe hepatocellular injury due to its highly toxic metabolite trichloromethyl free radical via the action of the cytochrome P450 system [71]. Intraperitoneal injection of CCl_4_ (50 *μ*L/kg) markedly induced liver injury in mice with increased serum levels of AST and ALT and upregulated IL-1*β*, TNF-*α*, iNOS, COX-2, and NF-*κ*B in hepatic tissues. Besides, CCl_4_ increased the MDA and decreased the GSH, SOD, GST, CAT, and GPx in liver tissues. Interestingly, pretreatment with PD (25-100 mg/kg/day) for 5 days before CCl_4_ injection can improve the liver injury via upregulation of transforming growth factor-beta1 (TGF-*β*1) in the liver tissues [72].

Almost all organ systems including humans and animals could be affected by arsenic (As), as could cause several hazardous effects on animals and humans via inducing oxidative stress [73, 74]. Previous researches have shown that PD treatment could ameliorate the As-induced histopathological damage in tissues, lipid peroxidation, and DNA damage in rats [75]. Another heavy metal named Cd also has a high toxic potential for humans and animals, and long-term exposure in Cd would result in serious damage in liver [76]. Treatment with PD (120 mg/kg) significantly increased the liver total oxidant status (TOS) and decreased the MDA in liver tissue of mice exposed in Cd [77].

Cholestasis might be induced by intrahepatic and systemic retention of toxic hydrophobic bile salts. Cholestatic liver diseases can develop into periportal inflammation, liver fibrosis, cirrhosis, and even hepatic failure [78, 79]. The toxicity of hydrophobic bile salt exposure in the liver can induce oxidative stress, subsequently leading to apoptosis and inflammatory necrosis [80]. PD could improve SOD activity, reduce MDA and serum AST, ALT, ALP, total bile acid (TBA), and total bilirubin (TBIL) levels, and inhibit ER stress, p-elf2*α*, CHOP, and hepatocellular apoptosis in the cholestatic mice induced by alpha-naphthylisothiocyanate (ANIT) and bile duct ligation (BDL). The results suggest that PD may alleviate cholestatic liver damage by inhibition of oxidative stress, ER stress, and apoptosis [81].

Fulminant hepatic failure (FHF) is characterized by overwhelming hepatic injury with failure of hepatocyte function, resulting in a devastating clinical syndrome of hepatic encephalopathy, severe coagulopathy, jaundice, and hydroperitoneum [82, 83]. Pretreatment with PD (10-100 mg/kg) could decrease the mortality of lipopolysaccharide/D-galactosamine- (LPS/D-GaIN-) induced FHF mice by alleviating liver damage and reducing AST and ALT. Furthermore, PD could also inhibit the TNF-*α*, endothelial cell adhesion molecule-1 (ECAM-l), intercellular cell adhesion molecule-1 (ICAM-l), NF-*κ*B, and myeloperoxidase (MPO) activities induced by LPS [84].

### 2.4. Polydatin and Liver Fibrosis

**Figure 5:**
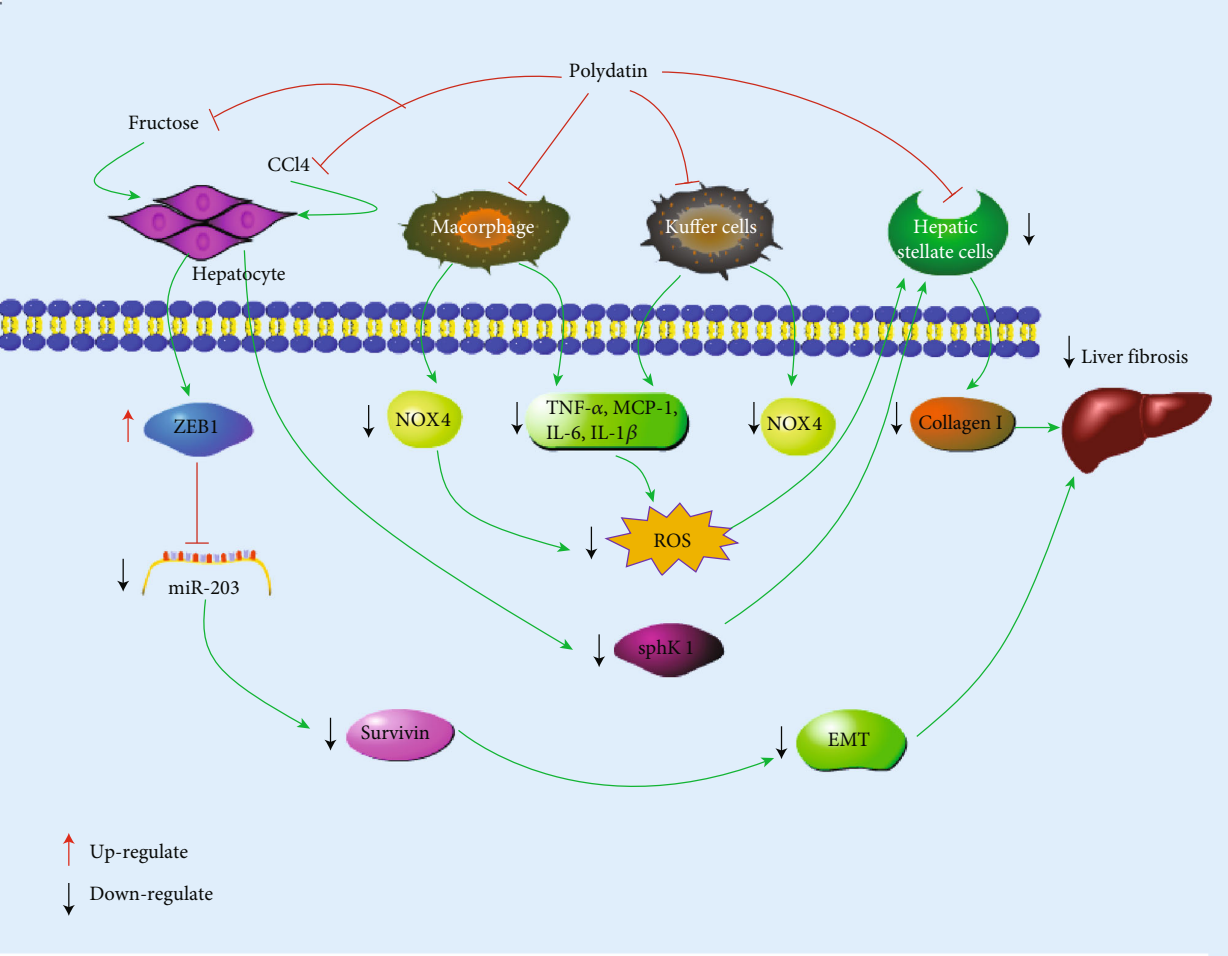
Cellular and molecular mechanisms of PD in the prevention of oxidative-associated liver fibrosis.

It is reported that uncontrolled simple steatosis might develop to some serious liver diseases such as hepatitis, fibrosis, and cirrhosis [64, 85]. During liver fibrosis, a mass of cellular and molecular events participates in the complex pathological process (Figure 5). Hepatocyte injury is the initial event in response to continuous wounding stimulation [86]. Besides, hepatic stellate cells (HSCs) have been considered playing a vital role in course of liver fibrosis [87, 88]. After a chronic liver damage, HSCs are activated and proliferated and then developed to a myofibroblastic phenotype with upregulated *α*-smooth muscle actin (*α*-SMA) that synthesizes ECM proteins, such as type I collagen [89]. However, PD treatment for 3-6 weeks could remarkably downregulate the *α*-SMA and suppress the increased collagen I and hydroxyproline, an amino acid contained in collagen in liver of mice [91]. Sphingosine kinase 1 (SphK1) plays critical roles in the activation of HSCs and liver fibrosis [92], and SphK1 was strongly induced in mice exposed to CCl_4_. It is reported that PD could attenuate the proliferation and activation of HSCs via inhibiting SphK1 signaling pathway in CCl_4_-induced mice, contributing to the suppression of liver fibrosis [95]. Using human immortalized HSC line of LX-2 induced by the platelet-derived growth factor-BB (PDGF-BB) or adenovirus-SphK1, it is reported that PD attenuated the collagen synthesis and apoptosis of hepatocyte and showed significantly antiproliferative effect against HSCs induced by PDGF-BB. Epithelial-mesenchymal transition (EMT) is a crucial biological process for development of fibrosis, and TGF-*β*1 signaling is one of the most critical profibrotic pathways [97, 98]. Zhao et al. reported that PD treatment (7.5, 15, and 30 mg/kg) for 11 weeks antagonized the nuclear translocation of the Zinc finger E-box binding homeobox 1 (ZEB1) and inhibit survivin-activated TGF-*β*1/Smad signaling, which was consistent with its protective effect on fructose-induced EMT and liver fibrosis. Inhibiting the nuclear translocation of ZEB1 by PD may be a new strategy to ameliorate EMT of liver fibrosis associated with high-fructose diet [99].

However, liver fibrosis was a complex pathophysiological process. If therapeutic methods are only aimed at decreasing the activation of HSCs, it often leads to undesirable outcomes [97]. Liver fibrosis is involving the mutual interaction between parenchymal hepatocytes and nonparenchymal liver cells, including HSCs, Kupffer cells (KCs), and macrophages [94, 95]. The activated KCs constitute a central component of the inflammatory response in liver fibrosis by producing amount proinflammatory cytokines such as MCP-1, TNF-*α*, and TGF-*β*1 and mediators related to oxidative stress that induce quiescent HSCs to differentiate into activated myofibroblasts, the principle ECM-synthesizing cells which play as the vital executor in hepatic fibrogenesis. In return, the activated HSCs promote the recruitment of macrophages from the bone marrow to augment the already-large number of KCs, further aggravating the deterioration of inflammation and fibrogenesis [90, 96]. Both in vivo and in vitro results suggested that polydatin-loaded-micelle (PD-MC) could remarkably decrease liver cell apoptosis and avoid HSCs and macrophage activation and inhibit inflammatory response by suppressing the activation of TLR4/NF-*κ*B p65 signaling and proinflammatory cytokines secretion in macrophages and oxidative stress [97].

### 2.5. Polydatin and Hepatocellular Carcinoma

Cancers are the leading killers for human being in the world, especially for aged people over than 55 years old. Besides surgery, chemotherapy remains the best choice for treating various cancers. In recent years, increasing scientific evidences have suggested that natural agents are precious resources for finding more novel and safe candidate drugs for treating cancers, including hepatocellular carcinoma (HCC), lung cancer, and breast cancer [100, 101]. Patients with chronic liver diseases and cirrhosis might result in HCC, a common primary malignancy in the liver. HCC is the third leading cause of cancer-related deaths in the world [94]. Unfortunately, the currently available chemotherapeutic agents are not practical for the treatment of advanced HCC [101–103]. In this regard, it is necessary to develop more effective compounds, which may provide a novel therapy for HCC treatment, especially in the advanced stage. PD can promote SMMC-7721 and HepG2 cell apoptosis via upregulating Bax/Bcl-2 ratio and suppressing proliferation by decreasing the Wnt/*β*-catenin signaling in hepatocellular carcinoma. The invasion and migration of cancer cells are believed to promote the metastasis of cancer to a large extent. HCC cell invasion and migration invasion assay and wound healing assay were suppressed by treatment with PD [104]. Therefore, PD might be a promising natural small molecule drug for early liver cancer treatment.

## 3. Clinical Reports and Toxicity Studies

In clinical practice, there was no research published on PD single used for treating liver diseases. However, lots of Chinese patent medicines, such as *Yi-du-Tiao-gan* mixture, liver Corelle tablet, and *Hu-gan-ning* capsule, have been used in clinical practice for the treatment of liver disease in China. In these Chinese patent medicines, *P. cuspidatum* was one of the primary medicinal materials and PD was one of the main active ingredients [105–107]. Only one team studied the clinical efficacy of PD in treating coronary heart disease in the elderly. The results illustrated that the effective of experimental group treatment rate was 91.67%, dramatically higher than that of the control group, 76.67%. The effectiveness of PD to treat elderly coronary heart disease was definite [108]. Besides, PD injection, applied in treating myocardial ischemia, cerebral ischemia, shock, and other cardiovascular and cerebrovascular diseases, has been approved to enter phase II clinical trials by the US Food and Drug Administration [109].

PD has a favorable safety profile in animals (up to a dose of 200 mg/kg) and was well tolerated in humans (40 mg twice a day for 90 days in phase II clinical trial). The safe evaluation was completed by the New Drugs Safety Evaluation and Research Center in the Chinese Academy of Medical Sciences, which demonstrated that no significant toxic effect existed after intravenous injection of PD for 30 days [110]. Another research reported that the LD_50_ of PD was (1000 ± 57.3) mg/kg injected intraperitoneally [111]. However, few researches are assessing the adverse reactions of PD in liver diseases. Only in one randomized clinical trial PD has been shown to exert a marked effect on abdominal pain in patients with irritable bowel syndrome through dietary supplementation. Peritonitis, some liver cell necrosis, and bone marrow fat hyperplasia would occur in varying degrees when intraperitoneal injection concentrations of 50, 150, and 700 mg/kg of PD for 42 days [112]. Gavaged with the maximum concentration and the maximum gavage volume of PD to mice, the survival rate of mice was 100%, and the accumulated maximum tolerable dose (MTD) amount per day is 75.5 g/kg. The IC_50_ of PD for human normal liver cells L02 is 263.05 *μ*g/mL [113].

## 4. Conclusion and Perspectives

Collectively, according to the abovementioned effects and mechanisms of polydatin (PD) on liver diseases, it is highly suggested PD is an effective natural product for treating oxidative stress-associated liver diseases, including alcoholic liver diseases, nonalcoholic liver diseases, liver injury, liver fibrosis, and hepatocellular carcinoma. Experimental evidence indicated that PD exhibits curative effect against liver diseases through various signaling pathways, such as PI3K/Akt/Nrf2/HO-1 and Sirt1/Nrf2, PI3K/Akt/mTOR. Overall, this review highlights the potential application of PD as a potential agent against liver diseases.

In past decades, lots of researches have focused on the preclinical therapeutical effects of PD against various liver diseases. However, the detail molecular mechanisms were not thoroughly explored. Modern research methods such as genomics, metabolomics, proteomics, and metagenomics can be used to conduct more in-depth investigations on the corresponding mechanisms of PD on liver diseases, which provide strong support and theoretical basis for the subsequent clinical research and ultimately developed this natural compound to a new drug for liver disorders [114–116]. Secondly, although researchers have concentrated on LD_50_ of PD, the study of reproductive, carcinogenic, and teratogenic toxicity was not involved. PD has a higher bioavailability than resveratrol, but its concentration in liver tissue was relatively low; intravenous injection of 20 mg/kg of PD, the maximum concentration in liver is 5.22 g ± 0.46 *μ*g/kg; for oral administration of 50 mg/kg of PD, the maximum concentration in liver is 4.47 ± 2.51 *μ*g/kg [117, 118]. Recently, it is suggested that the targeted drug delivery systems based on microenvironment sensitive polymeric nanocarriers had great potentials to increase the drugs' bioavailability, improving the therapeutic efficacy and minimizing the drug side effects [119]. It is necessary to find or synthesize related biopharmaceutical materials corresponding the characteristics of liver diseases to prepare new dosage forms of PD to ameliorate the therapeutic efficacy [120]. Therefore, potential safety hazard and restricted efficacy of PD remain to conquer for further clinical practices [96]. Finally, PD and curcumin have similar pharmacological effects on oxidative stress associated with liver disorders [4]. Will combination of PD and curcumin shows a more substantial therapeutic effect on liver diseases? It is worth to be further studied.

## Figures and Tables

**Table 1:** The contents of PD in different plants.

Plant	Content (*μ*g/g)	References
*Polygonum* *cuspidatum*	14430	[17]
Grape peel	11.22 − 11.65	[18]
Peanuts	0.22 − 1.44	[19]
*Polygoni* *Multiflori*	33.74	[20]
Mulberry	39.7 − 133.8	[21]
Cocoa products	Not mentioned	
Hop flowers	Not mentioned	
Soybeans	Not mentioned	

**Table 2:** Effects of polydatin in the protection and treatment of oxidative stress-associated liver diseases.

Liver disease type	Experimental model			Dose and formulation	Duration of treatment	References
Alcohol liver diseases	Animals	Male Wistar rats	Ethanol/7 mL/kg/every 12 h/(i.g.)	25, 50, and 100 mg/kg/day/(i.g.)	Pretreatment for 7 days	[41]
Hepatic steatosis	Animals	Zebrafish strain	Ethanol/350 mM (2% EtOH)/32 h at 28.5°C	6.25, 12.5, 25 *μ*g/mL	48 h	[42]
Acute liver injury	Animals	C57BL/6 male mice	Ethanol/50%/10 mL/kg/oral/2 days	50 and 100 mg/kg/day (i.g.)	Pretreatment for 8 days	[46]
Nonalcohol fatty liver	Animals	Male Sprague Dawley rats	High-fat diet/12 weeks	0.3%/day (i.g.)	12 weeks	[55]
Nonalcohol fatty liver	Animals	Male Sprague Dawley rats	Fructose-induced/drinking 10% Fructose/6 weeks	7.5, 15, 30 mg/kg (i.g.)	7 weeks	[56]
	Cells	BRL-3A/HepG2	4.5 mg/mL glucose/12 h	10, 20, and 40 *μ*M	24 h	
Nonalcoholic steatohepatitis	Animals	C57BL/6 male mice	Methionine-choline deficient diet/4 weeks	5 mg/kg (i.p.)	4 weeks	[59]
	Cells	HepG2 cells	250 *μ*M palmitic acid/24 h	5, 10, and 20 *μ*M	24 h	
Nonalcohol fatty liver	Animals	Male Sprague Dawley rats	High-fat diet/16 weeks	30, 90 mg/kg/day/(i.g.)	8 weeks	[60]
Nonalcohol fatty liver	Animals	Male C57/BL6 mice	High-fat diet/14 weeks	100 mg/kg/day/(i.g.)	4 weeks	[61]
NASH	Animals	C57Bl/KsJ-db/db (db/db) mice	Methionine-choline deficient/4 weeks	100 mg/kg/(i.g.)	Every other day for 4 weeks	[63]
	Cells	L02 cells	Palmitic acid/60 *μ*g/mL/24 h	24 *μ*M	24 h	
Liver injury	Animals	Male ICR mice	APAP/220 mg. kg^−1^/i.p.	25, 50, and 100 mg/kg/day/(i.g.)	Pretreatment for 7 days	[66]
Liver injury	Animals	Male Wistar albino rats	Cis/7 mg/kg/i.p.	25, 50, and 100 mg/kg/day/(i.g.)	Pretreatment for 10 days	[67]
Liver injury	Animals	Male ICR mice	Sulfur mustard/40 mg/kg/i.p.	100, 200, and 400 mg/kg/day	7 days	[70]
	Cells	L02 cells	Sulfur mustard/50 *μ*M/30 min	50 *μ*M	24 h	
Liver injury	Animals	Male ICR mice	CCl_4_/5 *μ*L/kg/i.p.	25, 50, and 100 mg/kg/day/(i.g.)	Pretreatment for 5 days	[72]
Liver injury	Animals	Male Wistar albino rats	As/100 mg/L/drinking	50, 100, and 200 mg/kg/day/(i.g.)	60 days	[75]
Liver injury	Animals	Male Wistar albino rats	Cadmium chloride/5 mg/kg/gastric gavage/4 weeks	120 mg/kg/day/(i.g.)	4 weeks	[77]
Fulminant hepatic failure	Animals	Balblc mice	LPS (50 *μ*g/kg) and D − GaIN (700 mg/kg)/i.p.	10, 30, 100 mg/kg/day/i.p.	Pretreatment for 1 h	[84]
Liver injury	Animals	Male C57BL/6 mice	ANIT/60 mg/kg/48 h (i.g.)	40, 60, and 80 mg/kg/day/(i.g.)	Pretreatment for 7 days	[90]
Liver fibrosis	Animals	C57BL/6 mice	CCl_4_/5 ml/kg/i.p./twice a week for 6 weeks	5 mg/kg/(i.p.)	3 and 6 weeks	[91]
Liver fibrosis	Animals	C57BL/6 mice	CCl_4_/50 *μ*L/kg/i.p./twice a week for 6 weeks	5 mg/kg/(i.p.)	6 weeks	[93]
	Cells	LX − 2 cells	PDGF − BB/10 ng/mL	10 *μ*M	24 h	
Liver fibrosis	Animals	Male Sprague Dawley rats	Fructose/10%/6 weeks/(i.g.)	7.5, 15, and 30 mg/kg/(i.g.)	11 weeks	[99]
	Cells	BRL − 3A cells	Fructose/5 mM	10, 20, and 40 *μ*M	6, 12, 24 h	
Hepatocellular carcinoma	Animals	Male BALB/c nude mice	HepG2 cells/5 × 10^6^/subcutaneous injection/120 mm^3^	25, 50, and 100 mg/kg/100 *μ*L (i.p.)	20 days	[104]
	Cells	HepG2and SMMC − 7721		1, 3, 10, 30, and 100 mM	48 h	

## Abbreviations

*α*-SMA: *α*-Smooth muscle actin
AGEs: Advanced glycation end products
ADH: Alcohol dehydrogenase
ALB: Albumin
ALDs: Alcohol liver diseases
ALDH: Aldehyde dehydrogenase
ALP: Alkaline phosphatase
ALT: Alanine aminotransferase
ANIT: Alpha-naphthylisothiocyanate
APAP: Acetaminophen
ASC: Apoptosis-associated speck-like protein
As: Arsenic
AST: Aspartate aminotransferase
Bax: BCL-2-associated
BDL: Bile duct ligation
Bcl-2: B-cell lymphoma-2
CAT: Catalase
CD: Cadmium chlorine
CHOP: C/EBP homologous protein
CYP2E1: Cytochrome P4502E1
CPT-1: Carnitine palmitoyl transferase-1
Cyt-C: Cytochrome c
ECAM-l: Endothelial cell adhesion molecule-1
ECM: Extracellular matrix
EMT: Epithelial-mesenchymal transition
ER: Endoplasmic reticulum stress
FASN: Fatty acid synthase
FFA: Free fatty acid
FHF: Fulminant hepatic failure
GADD45*α*a: Growth arrest and DNA damage-inducible gene, 45*α*a
GPx: Glutathione peroxidase
GR: Glutathione reductase
GSH: Glutathione
H_2_O_2_: Hydrogen peroxide
HCC: Hepatocellular carcinoma
HSCs: Hepatic stellate cells
HO-1: Heme oxygenase-1
IL-6: Interleukin-6
IL-1*β*: Interleukin-1*β*
ICAM-1: Intercellular cell adhesion molecule-1
KCs: Kupffer cells
LDH: Lactate dehydrogenase
LX-2: HSC line
MCD: Methionine-and choline-deficient
MMPs: Matrix metalloproteinases
LPS: Lipopolysaccharide
MCP-1: Monocyte chemoattractant protein-1
MDA: Malondialdehyde
mTOR: Phosphoinosmde-3-kinase/the mammalian target of rapamycin
MTD: Maximum tolerable dose
MPO: Myeloperoxidase
NADPH: Nicotinamide adenine dinucleotide phosphate oxidative (NOX)
NAFLDs: Nonalcoholic liver diseases
NASH: Nonalcoholic steatohepatitis
NF-*κ*B: Nuclear factor kappa B
NLRP3: The NOD-like receptor family, pyrin domain containing 3
NQO1: NAD(P)H, quinone oxidoreductase-1
NO: Nitric oxide
NOX: Nicotinamide adenine dinucleotide phosphate oxidative
Nrf2: Nuclear factor erythroid 2-related factor 2
PD: Polydatin
PDGF-BB: Platelet-derived growth factor-BB
PD-MC: Polydatin-loaded-micelle
PI3K: Phosphatidylinositol 3-kinase
PPAR-*α*: Peroxisome proliferator activated receptor-*α*
RNS: Reactive nitrogen species
ROS: Reactive oxygen species
SCD-1: Stearoyl-CoA desaturase-1
Sirt1: Sirtuin-1
SM: Sulfur mustard
SOD: Superoxide dismutase
SphK1: Sphingosine kinase 1
SREBP-1: Sterol regulatory element binging protein 1
TBA: Total bile acid
TBIL: Total bilirubin
TC: Total cholesterol
TFEB: Transcription factor EB
TG: Triglyceride
TGF-*β*1: Transforming growth factor-beta1
TLR4: Toll-like receptor 4
TNF-*α*: Tumor necrosis factor-*α*
TOS: Total oxidant status
TXNIP: Thioredoxin-interacting protein
ZEB1: Zinc finger E-box binding homeobox 1.
